# Characterization and comparative profiling of the small RNA transcriptomes in two phases of flowering in *Cymbidium ensifolium*

**DOI:** 10.1186/s12864-015-1764-1

**Published:** 2015-08-20

**Authors:** Xiaobai Li, Feng Jin, Liang Jin, Aaron Jackson, Xiang Ma, Xiaoli Shu, Dianxing Wu, Guoqiang Jin

**Affiliations:** Zhejiang Academy of Agricultural Sciences, Shiqiao Road 139, Hangzhou, 310021 People’s Republic of China; Hubei University, College of Life Sciences, Wuhan, 430062 People’s Republic of China; USDA-ARS, Dale Bumpers National Rice Research Center, Stuttgart, Arkansas 72160 USA; Shanghai Institutes for Biological Sciences, Chinese Academy of Sciences, Shanghai, 200031 People’s Republic of China; International Atomic Energy Agency Collaborating Center, Zhejiang University, Hangzhou, 310029 Peoples Republic of China; Agricultural Bureau of Yuhang District, Yuhang, Hangzhou, Peoples Republic of China

## Abstract

**Background:**

*Cymbidium ensifolium* is one of the most important ornamental flowers in China, with an elegant shape, beautiful appearance, and a fragrant aroma. Its unique flower shape has long attracted scientists. MicroRNAs (miRNAs) are critical regulators in plant development and physiology, including floral development. However, to date, few studies have examined miRNAs in *C. ensifolium*.

**Results:**

In this study, we employed Solexa technology to sequence four small RNA libraries from two flowering phases to identify miRNAs related to floral development. We identified 48 mature conserved miRNA and 71 precursors. These conserved miRNA belonged to 20 families. We also identified 45 novel miRNA which includes 21 putative novel miRNAs*, and 28 hairpin forming precursors. Two *trans*-acting small interfering RNAs (ta-siRNAs) were identified, one of which was homologous to TAS3a1. TAS3a1 belongs to the TAS3 family, which has been previously reported to target auxin response factors (ARF) and be involved in plant growth and floral development. Moreover, we built a *C. ensifolium* transctriptome database to identify genes targeted by miRNA, which resulted in 790 transcriptomic target unigenes. The target unigenes were annotated with information from the non-redundant (Nr), gene ontology database (GO), eukaryotic orthologous groups (KOGs) and Kyoto encyclopedia of genes and genomes (KEGG) database. The unigenes included MADS-box transcription factors targeted by miR156, miR172 and miR5179, and various hormone responding factors targeted by miR159. The MADS-box transcription factors are well known to determine the identity of flower organs and hormone responding factors involved in floral development. In expression analysis, three novel and four conserved miRNA were differentially expressed between two phases of flowering. The results were confirmed by RNA-seq and qRT-PCR. The differential expression of two miRNA, miR160 and miR396, targeted ARFs and growth regulating factor (GRF), respectively. However, most of these small RNA were clustered in the uncharacterized group, which suggests there may be many novel small non-coding RNAs yet to be discovered.

**Conclusion:**

Our study provides a diverse set of miRNAs related to cymbidium floral development and serves as a useful resource for investigating miRNA-mediated regulatory mechanisms of floral development.

**Electronic supplementary material:**

The online version of this article (doi:10.1186/s12864-015-1764-1) contains supplementary material, which is available to authorized users.

## Background

*Cymbidium ensifolium*, is a diploid plant with 40 chromosomes (2n) and an estimated haploid genome size of 4,000 Mb [1]. *C. ensifolium*, is under subgenus *Jensoa* of genus *Cymbidium* in the orchid family (Orchidaceae). *C. ensifolium* has a long juvenile phase before flowering, generally five to six years, which delays breeding programs [2]. The floral morphology is one of the most important factors determining its ornamental value. Thus, it will be necessary to understand the genetic mechanisms underlying floral development in *C. ensifolium* to change flowering time and modify floral traits. Although much effort has been devoted to the cloning and identification of key genes involved in floral development and flowering of *Cymbidium* species [3, 4], the role of non-coding RNAs in *C. ensifolium* floral development is poorly understood.

In general, small, non-coding RNAs are grouped into two major classes: microRNAs (miRNAs) and short-interfering RNAs (siRNAs). Both small RNA classes have the same chemical composition and mechanism of action. However, siRNAs and miRNAs can be distinguished by their origin, evolutionary conservation and the types of genes that they silence [5]. MiRNAs are endogenous non-coding RNAs of ~21 nucleotides (nt) in length, derived from single-stranded RNA hairpin precursors cleaved by a double-stranded-specific RNase (Dicer) in animals and Dicer-like1 (DCL1) in plants [6]. After DCL1 trims the hairpin precursor, the miRNA/miRNA* duplex is released, and most of miRNA* sequences are quickly degraded [7, 8]. However, some miRNA* occur in a large abundance, such as two conserved miRNA families, miR171 and miR396, which were identified in *Rosa hybrida* [9] and *Brassica napus* [10]. There also exist non-conserved or species-specific small interfering RNA in plants, which are the *trans*-acting small interfering RNAs (ta-siRNAs). Ta-siRNAs are similar to miRNAs and act *in trans* to regulate messenger RNAs (mRNAs) [11]. Ta-siRNAs were generated from TAS gene transcripts, which are cleaved by a miRNA, resulting in the production of small RNA fragments 21 nt in length that are in phase with the miRNA cleavage site [12].

MiRNAs play an essential role in plant flowering time and floral organ identity. In Arabidopsis, three miRNA families, miR156/ 157, miR159 and miR172, have been shown to be involved in flowering time control under special environmental conditions. The overexpression of miR156/157 can delay flowering significantly [13]. MiR159 can regulate floral transition in short-day photoperiods, by repressing the floral meristem-identity gene [14]. MiR172 can promote flowering by integrating ambient temperature signals into the flowering genetic network [15]. Similarly, in rice and maize, miR156 and miR172 have been shown to control the conversion of spikelet meristems to floral meristems to ensure the initiation of floral organ primordia [16, 17]. Moreover, miR156 and miR172 are also involved in floral organ formation. MiR172 controls the inner whorl organ formation [18], whereas the miR164 gene is involved in establishment of boundaries between components within floral organs, and regulating the resulting floral organs sizes [19]. Recently, there are a few studies on miRNA in other orchid genera, i.e. phalaenopsis [20], orchis [21], and erycina [22] but not in cymbidium.

Three major approaches have been used for identifying miRNAs in plants: forward genetics, bioinformatic prediction and direct cloning and sequencing [23]. Only a few miRNAs have been identified by forward genetic studies [24, 25] and bioinformatic prediction for species-specific miRNAs is difficult, especially for species without a well-defined genome or a reference genome. The development of high-throughput sequencing technologies has greatly improved the success of detecting miRNA’s through direct cloning and sequencing. The Solexa platform, which is capable of yielding a high number of reads up to 35 bp in size [26] is suitable for sequencing miRNA, and identifying low-abundance and tissue-specific miRNA [27].

In our study, we deep sequenced the small RNA transcriptome in *C. ensifolium u*sing Solexa sequencing. The short *C. ensifolium* sequences were used to predict conserved and novel miRNAs. In order to explore the potential genes targeted by miRNA, we also created a large *C. ensifolium* mRNA transcriptome for comparison. The objective of the present study was to discover miRNAs and their corresponding target genes involved with *C. ensifolium* floral development.

## Methods

### Sample collection and preparation

**Fig. 1:**
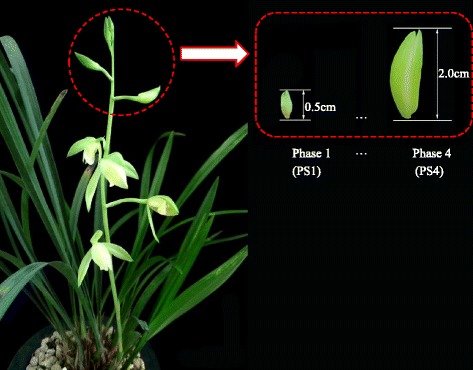
Developmental phases of the flower bud in *C. ensifolium*. Phase 1 (PS1), bud length < =0.5 cm, is the primary phase; Phase 4 (PS4), with bud length between 2.0 cm to 3.0 cm, is the final phase before blooming

Native cultivars of *C. ensifolium* “Tiegusu” with light green flowers were grown in a greenhouse under natural light with temperatures between 23 °C and 28 °C (Hangzhou, China). “Tiegusu” is one of the most widely known commercial cultivars in China. Flower buds were collected from two phases of floral development. In phase 1 (PS1) the bud dimension is < =0.5 cm; in phase 4 (PS4) bud dimension is between 2 cm and 3 cm in length (Fig. 1). Each tissue sample consisted of a mixture of five to eight flower buds. The two biological replicates for phase 1 and phase 2 resulted in four libraries.

### RNA isolation and quality control

Total RNA was isolated using a Trizol kit (Invitrogen, USA). Samples were run on a 1 % agarose gel to check for RNA degradation and contamination. RNA purity was checked using the NanoPhotometer® spectrophotometer (IMPLEN, CA, USA). RNA concentration was measured using Qubit® RNA Assay Kit in Qubit® 2.0 Flurometer (Life Technologies, CA, USA). RNA integrity was assessed using the RNA Nano 6000 Assay Kit of the Agilent Bioanalyzer 2100 system (Agilent Technologies, CA, USA).

### Library construction and small RNA sequencing

A total amount of 3 μg total RNA per sample was used as input material to construct small RNA libraries. Sequencing libraries were generated using NEBNext® Multiplex Small RNA Library Prep Set for Illumina® (NEB, USA.) following manufacturer’s recommendations and index codes were added to attribute sequences to each sample. Briefly, NEB 3’ SR adaptors were ligated to the 3' end of small RNA. After the 3' ligation reaction, the SR RT primer was hybridized to the 3' SR adaptor transforming the single-stranded DNA adaptor into a double-stranded DNA molecule. A 5´end adapter was then ligated to the 5´end and first strand cDNA was synthesized using M-MuLV Reverse Transcriptase (RNase H). PCR amplification was performed using LongAmp Taq 2X Master Mix, SR Primer for illumina and an index (X) primer. PCR products were purified on a 8 % polyacrylamide gel (100 V, 80 min). DNA fragments corresponding to 140 ~ 160 bp (the length of small noncoding RNA plus the 3' and 5' adaptors) were recovered and dissolved in 8 μL elution buffer. Library quality was assessed on the Agilent Bioanalyzer 2100 system using DNA High Sensitivity Chips. All of these processes were performed at the Beijing Novo Gene Genomics Institute, China.

The clustering of the index-coded samples was performed on a cBot Cluster Generation System using TruSeq SR Cluster Kit v3-cBot-HS (Illumina) according to the manufacturer’s instructions. After cluster generation, the library preparations were sequenced on an Illumina Hiseq 2500/2000 platform and 50 bp single-end reads were generated. The data were uploaded to NCBI (http://www.ncbi.nlm.nih.gov/) for public use (Accession: SRR2037124).

### Data filtering and read mapping

Reads with Poly N’s, 5’ adapter contaminants, low quality, or lacking the 3’ adapter or inset tags were removed. The Q20, Q30, and GC-content of the raw data were calculated and read lengths within a specific size range from 17 to 30 nt were chosen for analysis.

To remove tags originating from protein-coding genes, repeat sequences, ribosomal RNA (rRNA), transfer RNA (tRNA), small nuclear ribonucleic acid (snRNA), and small nucleolar RNA (snoRNA), small RNA tags were mapped to RepeatMasker, Rfam, and related databases of the model species, i.e. Oryza and Arabidopsis.

The small RNA tags were mapped to *C. ensifolium* transctriptome sequence [28] using Bowtie [29] and analyzed for their expression and distribution on the reference genome without mismatches present.

### Conserved miRNA alignment

Mapped small RNA tags were used to look for conserved miRNA. miRBase20.0 was used as a reference, modified software mirdeep2 [30] and srna-tools-cli [31] were used to identify potential miRNA and draw the secondary structures. Novo Custom scripts (Beijing Novo Gene Genomics Institute, China) were used to obtain the miRNA counts as well as base bias on the first position of identified miRNA with certain length and on each position of all identified miRNA respectively.

### Novel miRNA prediction

The characteristics of hairpin structure of miRNA precursor can be used to predict novel miRNA. The available software miREvo [32] and mirdeep2 [30] were integrated to predict novel miRNA through exploring the secondary structure by identifying the Dicer cleavage site and the minimum free energy of the small RNA tags unannotated in the former steps. Ta-siRNA was identified by comparing with ta-siRNA of Arabidopsis and rice using BLAST, or predicted by the software “UEA sRNA tools” [31]. Analysis of miRNA counts and base bias was performed as conserved miRNA.

### Small RNA annotation summary

During the alignment and annotation process, some small RNA tags may be mapped to more than one category. To make every unique small RNA map to only one annotation, we used the following priority rule: conserved miRNA > rRNA > tRNA > snRNA > snoRNA > repeat > novel miRNA > ta-siRNA.

### MiRNA family analysis, target gene prediction and annotation

In our analysis pipeline, conserved miRNA used miFam.dat (http://www.mirbase.org/ftp.shtml) to look for families, and novel miRNA precursors were submitted to Rfam (http://rfam.sanger.ac.uk/search/) to identify Rfam families. Target gene prediction of miRNA was performed by psRobot_tar in psRobot for plants [33]. The 5’-end of the cleavage product of *C. ensifolium* DEF3 (*Cen*DEF3) (JQ326260.1) was determined by a modified 5’-RACE using the RLM-RACE GeneRacerTM kit (Invitrogen, Carlsbad, CA, USA). The amplification was performed according to the manufacturer’s instructions. After reverse transcription, the 5’-end of the cleavage product was amplified using the GeneRace 5’ primer and gene specific reverse primers *Cen*DEF3R 5’-TCCCGCAGTAAGTTCCTGTGGGTTTC-3’. The amplified product was sequenced.

Target genes were compared with protein databases, including the non-redundant database (http://www.ncbi.nlm.nih.gov/), using BLASTX with a significance cut-off E-value of 1e-5. Gene Ontology (GO) enrichment analysis was performed on the target genes of differentially expressed miRNAs using GOseq R package [34]. GO terms with corrected P-value less than 0.05 were considered significantly enriched by target genes. GO terms with significance were summarized into major categories using GOSlim Viewer with the Plant GOSlim set. KEGG pathway analyses of the target genes were performed using the Kyoto Encyclopedia of Genes and Genomes (KEGG) (http://www.genome.ad.jp/kegg/) database. We used KOBAS [35] software to test the statistical enrichment of the target unigenes in KEGG pathways, with the threshold of corrected P value <0.05.

### Expression analysis of miRNA

miRNA expression levels were estimated by TPM (transcript per million) using the following criteria [36]. Normalized expression = mapped readcount/Total reads*1000000. Differential expression analysis of two flowering phases was performed using the DESeq R package (1.8.3). MiRNAs that had change ratios of more than 2 or less than 0.5 (Fold change Log2 > 1 or < −1) and P < 0.01 was set as the threshold for significant differential expression by default.

To validate the presence and expression of the identified miRNAs, miRNAs with differential expression and eight random miRNA were selected for stem-loop Real-time PCR (RT-qPCR) as described previously [37]. The total RNA was reverse-transcribed using miRNA specific stem-loop primers (Additional file 1). The reverse transcription reaction was performed with M-MLV (Takara, China), and the reverse-transcribed products were used as the template for RT-qPCR with gene-specific primers (Additional file 1). All reactions were assayed in three biological and technical replications, and performed in an ABI PRISM 7900HT (Applied Biosystems, USA) using Platinum SYBR Green qPCR SuperMix-UDG (Invitrogen, USA). PCR condition consisted of: pre-denaturation and hot start Taq activation at 95 °C for 5 min, then 40 cycles of 95 °C for 15 sec, and 60 °C for 30 sec. The U6 snRNA was used for normalization. The relative expression level of miRNA was calculated according to the method of Livak and Schmittgen [38]. In RT-qPCR, the significant threshold was set at the changes in ratios more than 2 or less than 0.5 (Fold change Log2 > 1 or < −1) and P < 0.01.

## Results

### sRNA profile in *C. ensifolium* floral buds

To identify novel miRNAs and investigate the effects of miRNAs during floral development in *C. ensifolium*, we constructed four small RNA libraries of the floral buds at phase 1 (PS1A, PS1B) and phase 4 (PS4A, PS4B) respectively. Deep sequencing of the small RNA libraries generated the mean number of raw reads, 10,372,041 and 9,105,582, for PS1 and PS4, respectively (Table 1). After removing adapter sequences and discarding low-quality reads, the mean number of clean reads, 10,131,741 and 8,911,915, for PS1 and PS4, respectively, remained for analysis. Finally, the reads ranging from 17 to 30 nt were selected for subsequent analysis. The selection resulted in the mean number of reads, 7,004,064 and 7,231,254, for PS1 and PS4, respectively (Table 2).

**Table 1:** Summary of small RNA in four libraries from two flowering phases

Sample	Total		Bases	Clean	
	Reads	Percent		Reads	Percent
PS1A	11,466,782	100.00 %	0.573G	11,156,921	97.30 %
PS1B	9,277,300	100.00 %	0.464G	9,106,560	98.16 %
PS4A	9,210,594	100.00 %	0.461G	8,969,985	97.39 %
PS4B	9,000,570	100.00 %	0.450G	8,853,844	98.37 %

Clean reads: Reads were obtained after removing Poly N’s, 5’ adapter contaminants, low quality reads, or reads lacking the 3’ adapter or inset tags

**Table 2:** Comparison between small RNA and the *Cymbidium* transcriptome

Sample	Total sRNA		Mapped sRNA		" + " Mapped sRNA		"-" Mapped sRNA	
	Reads	Percent	Reads	Percent	Reads	Percent	Reads	Percent
PS1A	5,629,134	(100.00 %)	2,807,112	(49.87 %)	2,044,383	(36.32 %)	762,729	(13.55 %)
PS1B	8,378,994	(100.00 %)	3,605,560	(43.03 %)	2,570,495	(30.68 %)	1,035,065	(12.35 %)
PS4A	6,512,965	(100.00 %)	2,456,095	(37.71 %)	1,692,808	(25.99 %)	763,287	(11.72 %)
PS4B	7,949,543	(100.00 %)	2,898,190	(36.46 %)	1,964,994	(24.72 %)	933,196	(11.74 %)

“+” Mapped sRNA: reads were mapped to reference sequences in the same orientation

“-” Mapped sRNA: reads were mapped to reference sequences in the reverse orientation

**Fig. 2:**
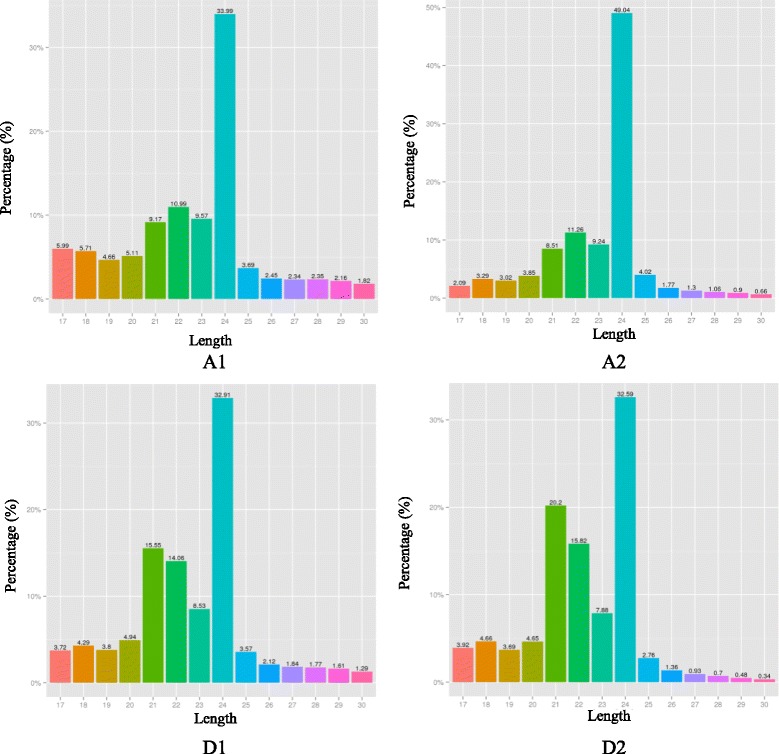
Length distribution of small RNA in four libraries derived from two *C. ensifolium* flowering phases. A1 and A2: two PS1 libraries from flowering phase I, D1 and D2: two PS1 libraries from flowering phase II. The unigenes are grouped from 17 nt to 30 nt with each column representing the number of unigenes of that specific length

The size distribution of small RNAs is summarized in Fig. 2. Overall sRNA ranging from 21 to 24 nt was common, and the 24 nt RNA was the most abundant. The 22-nt sRNAs was the second most abundant in PS1, while the 21 nt sRNAs were the second most abundant in PS4. Of these, almost half of the reads in two PS1 libraries, and one third of the reads in two PS4 libraries could be mapped to the *C. ensifolium* transcriptome (Table 2). However, a large percentage of small RNAs could not be mapped since the complete *C. ensifolium* genome sequence is not available.

The mapped small RNA sequences were clustered into several RNA classes, such as conserved miRNAs, rRNAs, tRNAs, snRNAs, snoRNAs, repeat and novel miRNA, TAS, and an uncharacterized group (Table 3). Most of these small RNA (89.45 %, group reads/the total reads*100 %) were clustered in the uncharacterized group. The second most abundant group (6.15 %) was rRNA. A search against miRbase20.0 identified 0.41 % conserved miRNAs, and 1.30 % novel miRNAs. The abundance of so many unknown sRNAs suggests that there may be many novel small non-coding RNAs yet to be discovered.

**Table 3:** The category of all small RNA in four libraries from two flowering phases

Types	Mean Reads	Mean Percent	Flowering Phase I				Flowering Phase IV			
			PS1A Reads	PS1A Percent	PS1B Reads	PS1B Percent	PS4A Reads	PS4A Percent	PS4B Reads	PS4B Percent
Total	2,941,739.25	100.00 %	2,807,112	100.00 %	3,605,560	100.00 %	2,456,095	100.00 %	2,898,190	100.00 %
conserved miRNA	11,935.75	0.41 %	8,417	0.30 %	14,261	0.40 %	13,059	0.53 %	12,006	0.41 %
rRNA	182,343.00	6.15 %	170,965	6.09 %	241,271	6.69 %	138,161	5.63 %	178,975	6.18 %
tRNA	14,702.75	0.54 %	33,385	1.19 %	3,937	0.11 %	18,195	0.74 %	3,294	0.11 %
snRNA	11,569.50	0.41 %	37,868	1.35 %	1,539	0.04 %	4,455	0.18 %	2,416	0.08 %
snoRNA	9,030.75	0.31 %	9,925	0.35 %	6,176	0.17 %	6,058	0.25 %	13,964	0.48 %
repeat	41,823.75	1.42 %	31,818	1.13 %	55,054	1.53 %	35,002	1.43 %	45,421	1.57 %
novel_miRNA	37,403.00	1.30 %	24,024	0.86 %	48,050	1.33 %	54,596	2.22 %	22,942	0.79 %
TAS	349.5	0.01 %	336	0.01 %	434	0.01 %	335	0.01 %	293	0.01 %
uncharacterized group	2,632,581.25	89.45 %	2,490,374	88.72 %	3,234,838	89.72 %	2,186,234	89.01 %	2,618,879	90.36 %

### Conserved miRNA, their nucleotide bias and family designations in *C. ensifolium*

To identify conserved miRNAs in *C. ensifolium*, all mapped small RNA reads in the transcriptome [28] were searched against known miRNAs in the database. The result showed that 47,743 total reads representing 667 unique sRNAs matched to conserved miRNAs (Table 4). In these libraries, two PS1 libraries had the mean of 11,339.00 (161.50 unique) reads, and two PS4 libraries had the mean of 12,532.50 (177.00 unique) reads. Of these unique sRNA, 48 mature miRNAs and 71 precursors were identified. All of precursors are able to adopt hairpin structures resembling the fold-back structure of a miRNA precursor.

**Table 4:** Summary of the conserved miRNA

Types	Total	Flowering Phase I			Flowering Phase IV		
		PS1A	PS1B	Mean	PS4A	PS4B	Mean
Mapped mature	48	38	40	39.00	39	39	39.00
Mapped hairpin	71	55	57	56.00	63	54	58.50
Mapped uniqe sRNA	677	167	156	161.50	179	175	177.00
Mapped total sRNA	47,743	8,417	14,261	11,339.00	13,059	12,006	12,532.50

**Fig. 3:**
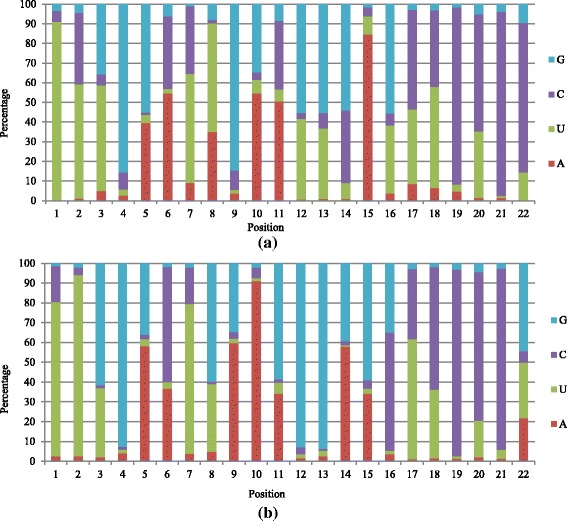
Examination of nucleotide bias within conserved (**a**) and novel miRNA (**b**) reads. Height of bar is proportional to the frequency of the corresponding base at the given position from 1st to 22nd

Analysis of nucleotide bias was performed on conserved miRNA. The analysis showed that uracil (U) appeared at the first three positions with a high frequency (Fig. 3a). At the 1st, 2nd and 3rd positions, the percentage of U averaged 90.99 %, 57.91 % and 53.73 % respectively. In contrast, guanine (G) or cytosine (C), were seldom present in the first two nucleotides. G and C were the most common nucleotides found in the last four base pair positions of miRNA. C occupied very high percentages, varying from 59.64 % to 90.19 %. By contrast, adenine (A) and uracil (U) were seldom seen near the end.

A total of 20 miRNA families were identified among these conserved miRNAs, and have been shown to be conserved across 66 plant species (Additional files 2 and 3). In *C. ensifolium*, the biggest family was miR166 with 10 members, followed by miR156 with 7 members, and miR171_1 and miR159 both with 6 members. Moreover, miR156/157 was the most popular family, and found in 51 plant species, followed by miR159/319, miR396, and miR166, which are found across 42 plant species. The miR171_1, and miR160 families were identified as well and occur in 40 and 39 different plant species, respectively. The abundance of conserved miRNA varied greatly. The majority of miRNAs had less than 10,000 transcripts per million (TPM) in our study, and some rare miRNAs had less than 100 TPM. Three miRNA, cen-miR159a.1, cen-miR166a-3p and cen-miR166m were very prevalent. Cen-miR159a.1 was the most abundant, followed by cen-miR166a-3p and cen-miR166m, and their TPM averaged 118,849.48, 60,627.64 and 60,627.64, respectively (Additional file 4).

### Novel miRNAs and their nucleotide bias in *C. ensifolium*

**Fig. 4:**
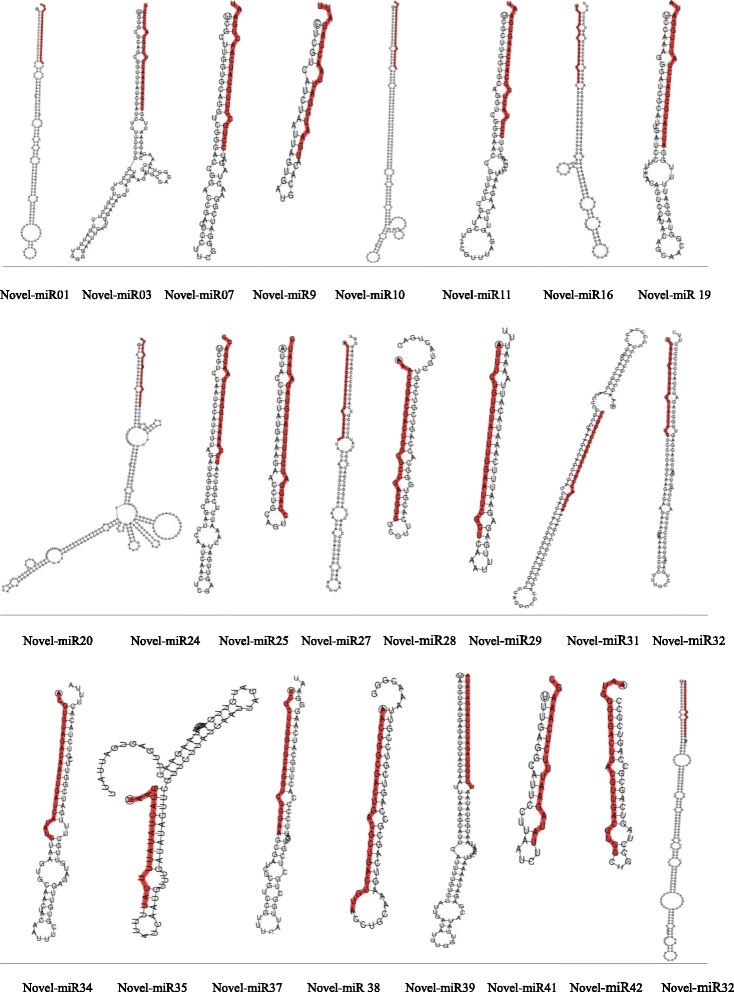
Predicted fold-back structures for the novel miRNAs in *C. ensifolium*. Mature miRNA sequence is shown in red

In addition to the conserved miRNA, we identified novel miRNAs in *C. ensifolium* floral buds. 149,612 novel miRNA reads were identified from four flower bud libraries, representing 1,627 unique miRNA (Table 5). The number of reads for these predicted novel miRNAs was more than that observed for conserved miRNAs. These unique sRNAs were derived from 45 novel miRNAs including 21 novel miRNA*. These novel miRNA were involved in 28 hairpin miRNA precursors. Precursors formed characteristic stem-loop structures (Fig. 4). The corresponding miRNA* for these novel families were predicted, providing more evidence that they are indeed novel miRNAs. In general, the miRNA had much more reads than miRNA*. For instance, the novel-miR01 and novel-miR44 were most abundant among novel miRNA families, whereas the corresponding miRNA* were relatively deficient. Interestingly, the novel-miR05* was the most common among miRNA*, while the counterpart was barely detected. The expression of novel miRNA was also tissue specific. For example, two novel miRNA, i.e. novel-miR16 and novel-miR20*, were found only at PS4 (Additional file 4).

**Table 5:** The summary of the novel miRNA

Types	Total	Flowering Phase 1			Flowering Phase 4		
		PS1A	PS1B	PS1 Mean	PS4A	PS4B	PS4 Mean
Novel mature miRNA	24	20	21	20.50	24	21	22.50
Novel miRNA*	21	14	13	13.50	16	14	15.00
Novel hairpin	28	24	25	24.50	28	25	26.50
Mapped uniqe sRNA	1,627	334	394	364.00	455	444	449.50
Mapped total sRNA	149,612	24,024	48,050	36,037.00	54,596	22,942	38,769.00

Novel miRNA was analyzed for nucleotide bias at each position. Uracil (U) appeared at the beginning of the miRNA with a high frequency (Fig. 3b). At the 1st and 2nd positions, the percentage of U averaged 77.86 % and 91.50 % respectively. Conversely, guanine (G) and cytosine (C) were seldom used as the first two nucleotides. Cytosine frequently occurred near the end, and varied from 62.14 % to 94.18 % from the 18th to 21st position. Conversely, adenine (A) and uracil (U) were seldom at the end. The obvious difference with conserved miRNA was that G and C account for almost half of the nucleotide occurrences at the 3rd and the last position.

Trans-acting siRNAs (ta-siRNAs) are a class of small RNAs that regulate plant development. We searched against TAS database and predicted Ta-siRNAs in *C. ensifolium*. The search resulted in two ta-siRNA genes, one of which is homologous to TAS3a1.

### Target prediction and functional analysis of miRNA in *C. ensifolium* floral buds

Due to the lack of genomic information available for *C. ensifolium*, we constructed a transcriptome database for predicting the miRNA targets [28]. A total of 47 conserved and one novel miRNAs targeted 790 potential unigenes, with an average of 16.81 per unigenes/miRNA (Additional file 5). Among the miRNA, cen-miR156k targeted the most unigenes (110), followed by cen-miR396a-3p (88), and cen-miR156a (75). cen-miR160e-5p and cen-miR166b-5p targeted two sequences, while cen-miR5538 targeted only one. Most of miRNA targeted multiple sites, indicating that a single miRNA can regulate more than one unigene. Conversely one unigene was also targeted by several miRNAs. A total of 162 unigenes could be regulated by more than one miRNA, two of which were targeted by 8 conserved miRNA.

**Fig. 5:**
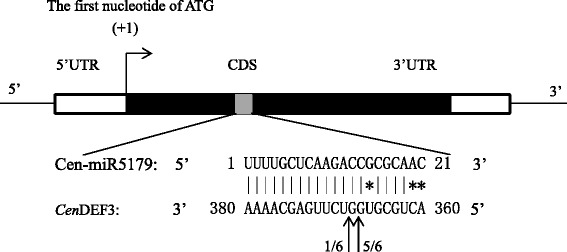
Cleavage of cen-miR5179 on *Cen*DEF3 in *C. ensifolium*. The corresponding region from the position 360 to 380, considering the first nucleotide of the ATG start codon as position (+1).The arrows indicate the position of the cleavage and the number of clones corresponding to each site as deduced by the cloning and sequencing of the obtained fragment

The 790 miRNA target unigenes were searched against the non-redundant database, 656 of which had hits that exceeded the E-value threshold (Additional file 5). Among 656 targets, 62 were involved in floral development/flowering time. Examples include squamosa promoter-binding-like protein (SPB) (cen-miR156a, cen-miR529b, and cen-miR156k), floral homeotic protein APETALA 2 (cen-miR172a and cen-miR172c), and AP3/MADS5/DEFICIENS-like MADS-box transcription factor (cen-miR156a and cen-miR5179). The cleavage product of cen-miR5179 on *C. ensifolium Cen*DEF3 transcript were successfully amplified, and miR5179 mediated cleavage was confirmed by modified 5’ RLM-RACE (Fig. 5). Additionally, 17 targets were involved in various hormone signaling pathways, such as auxin response factor, (targeted by cen-miR171a, cen-miR167d-5p, cen-miR167a-5p, cen-miR160e-5p, and cen-miR160a-5p), and ethylene responsive element binding protein 1 (targeted by cen-miR529b). However, 134 targets did not match any homologue in database. A large number of targets were only annotated with vague information, e.g. 95 targets were “unnamed protein”, and 93 were not predicted.

### Differential expression of miRNA between two *C. ensifolium* flowering phases and their target genes

**Fig. 6:**
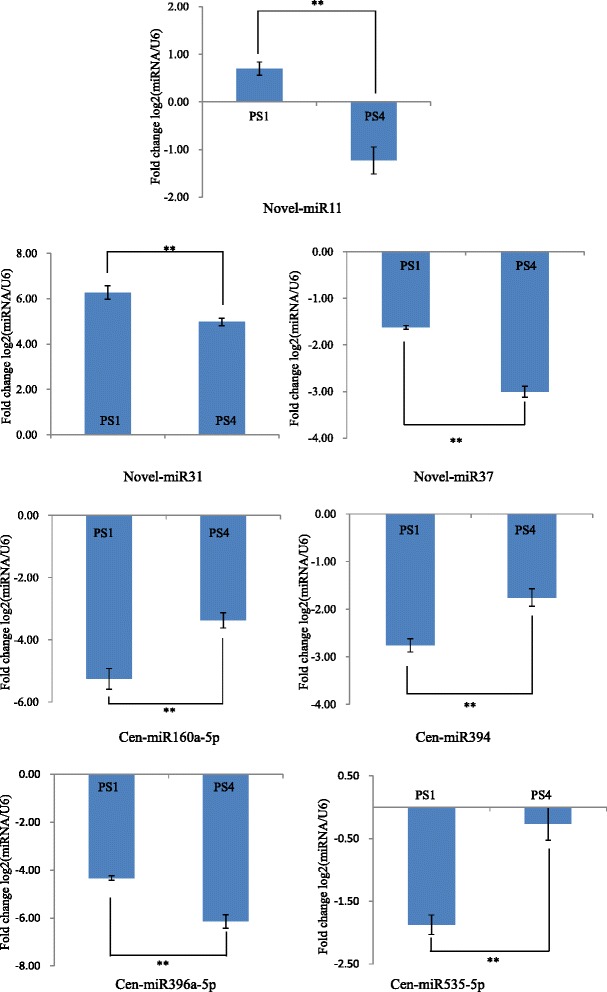
Expression levels of four conserved and three novel miRNAs between two flowering phases with significance in qRT-PCR. Based on RNA-seq results, 12 significantly different expressed miRNA were exposed to qRT-PCR assay for validation, which resulted in seven validated miRNA. Each experiment consisted of three biological and technical reps. The relative expression levels of the selected genes were calculated using the 2^–ΔΔCT^ method, and the significant threshold was set at 0.01 (**: <0.01). The U6 gene was used as a Control. Error bars represent the standard deviation of the mean expression values. At PS4, novel-miR11, novel-miR31, novel-miR37 and cen-miR396a-5p were down-regulated, while cen-miR160a-5p, cen-miR394 and cen-miR535-5p were up-regulated

To identify differential expression of miRNAs between two flowering phases, normalized miRNA levels were compared between PS1 and PS4. MiRNAs that had fold change log2 > 1 or < −1, and P < 0.01, were considered to be differentially expressed. Five novel and seven conserved miRNA sequences were differentially expressed. Based on the results of high-throughput sequencing, we performed qRT-PCR on these miRNA. In our qRT-PCR experiment, seven (58.33 %) of these miRNA exhibited significantly different expression (Fig. 6; Additional file 6). Among the seven miRNA, three miRNA were significantly up-regulated at PS4, while the other four miRNA were down-regulated at PS4. The expression patterns were similar to those detected by the RNA-Seq data (Pearson correlation coefficients = 0.948, P < 0.01) (Additional file 7). The results demonstrated the reliability of the mRNA-seq results. Thus, our experiments confirmed that the seven miRNAs were differentially expressed during two flowering phases.

**Fig. 7:**
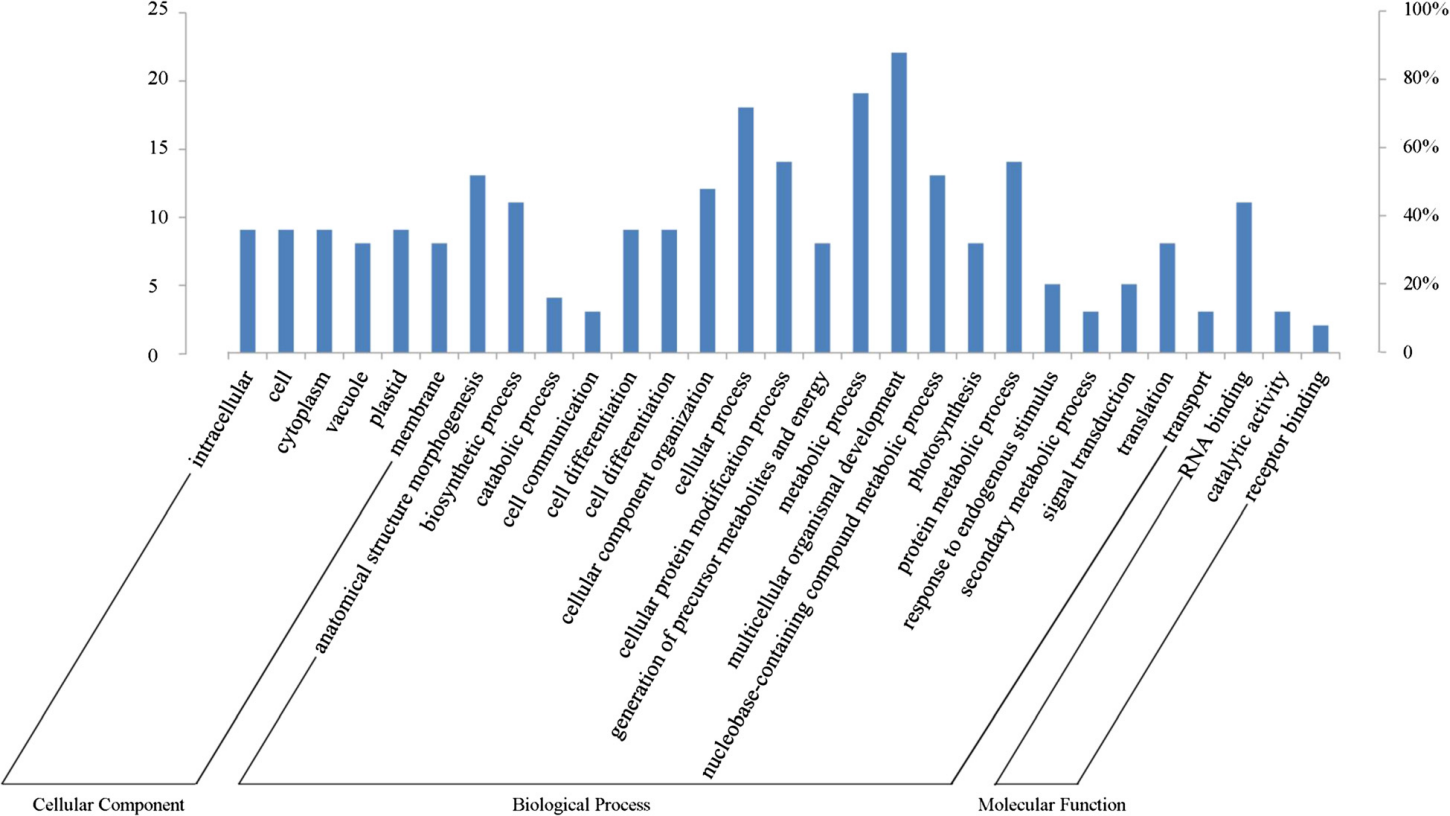
Gene ontology of unigenes targeted by differently expressed miRNAs. The significantly enriched GO terms were categorized according to cellular component, molecular function and biological process

**Fig. 8:**
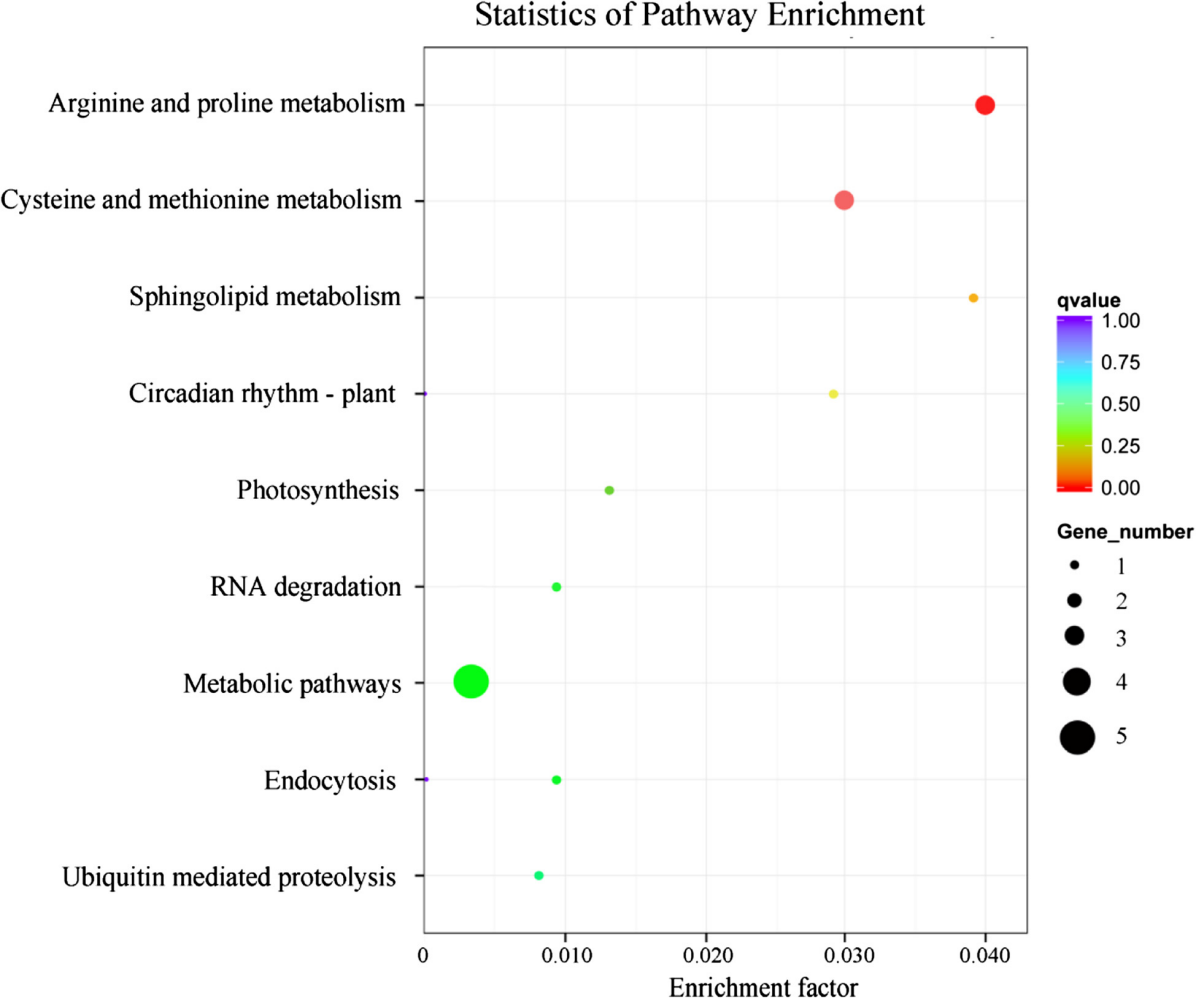
The nine enriched KEGG pathways of the target genes. The genes were targeted by differentially expressed miRNAs between floral development phases in *C. ensifolium*. The enrichment factor is on the x-axis and the y-axis contains the pathway names. The size of each point represents the number of genes enriched in a particular pathway. A larger enrichment factor value and lower Q-values indicates a greater degree of enrichment

The annotation of the target unigene of differentially expressed miRNAs was conducted based on GO enrichment and KEGG analyses. GO categories were assigned for target unigene, and 50 putative targets were assigned into 527 GO terms (Additional file 8). A total of 164 significant GO terms (Corrected P value < 0.05) were categorized as “biological process”, “cellular component” and “molecular function” (Fig. 7). Within the biological process category, the unigenes were classified into 20 groups, of which the most represented GO terms were related to “multicellular organismal development”, followed by “metabolic process” and “cellular process”. Based on molecular function, the unigenes were classified into three groups, of which the top one was “RNA binding”. Based on cellular components, the unigenes were classified into six groups, of which all were similarly represented. KEGG pathway analysis was performed for biological interpretation of these target unigenes of differentially expressed miRNAs. A total of nine biochemical pathways were involved with the miRNAs target unigenes (Additional file 9). On the threshold of corrected P-value <0.05, two were significantly enriched pathways involved with six unigenes, i.e. “Arginine and proline metabolism” and “Cysteine and methionine metabolism” (Fig. 8).

## Discussion

### Distribution and nucleotide bias of sRNA in *C. ensifolium* floral buds

The sRNAs ranging from 21 to 24 nt were common in this study. These sRNA represent the typical plant mature miRNAs [39]. The majority of 24 nt sRNA is congruent with previous reports on Arabidopsis [40], Oryza [41], Arachis [42], Medicago [39], Populus [43], Gossypium [44, 45], Phalaenopsis [20] and Orchis [21]. However, the size distribution differed from wheat and conifer small RNA obtained through 454 high-throughput sequencing [46, 47] and Chinese yew small RNA obtained through Solexa technology [48]. In conifers, 24 nt miRNAs comprise only a small percentage of sRNA, possibly due to the lack of DCL3, the enzyme that cleaves the precursor into mature 24 nt RNAs in angiosperms [46, 49]. In the current study the main difference between PS1 and PS4 was seen within the 21 and 22 nt sRNAs.

In analysis of nucleotide bias, uridine (U) dominated the first position of the novel and conserved miRNAs in this study. The result was consistent with previous studies [50]. The 5′ terminal nucleotide depends on an miRNAs biogenesis mechanism, for example, AGO1 (a protein from Dicer) harbors miRNAs with a 5’ terminal U [50]. The difference between conserved and novel miRNA, was that G/C accounted for almost 60 % at the 3rd base from the 5’ end in novel miRNA and almost 80 % at the last positions in conserved miRNA. The region from 2 to 8 is the “seed region” of an miRNA, which binds with the target gene(s) during gene regulation [51]. The difference in this region suggested conserved and novel miRNA might target different mRNA or different positions on the same mRNA. miRNAs targeting different positions is correlated with different silencing activity, for example, lower GC content was correlated with higher activity [50].

### Specific expression of conserved miRNA in *C. ensifolium* flower buds

The expression of miRNA families differs across species (Additional files 2 and 3). In *C. ensifolium,* cen-miR159a.1 was the most abundant, followed by cen-miR166a-3p and cen-miR166m. In previous studies on other plant species tae-miR169b in wheat, osa-miR169 in rice, zma-miR156a in maize [52] and mtr-miR156a in *Medicago truncatula* [53] are the most frequently observed miRNAs, while miR156 in rice and wheat exhibits low abundance [47]. This may suggest a tissue-specific or species-specific expression profile for miRNAs. The highly expressed miRNAs may be involved in regulating essential functions. For example, miR159 is associated with flowering in short days and male sterility (Palatnik et al. 2007; Achard et al. 2004; Quesada et al. 2005). MiR166 targets genes that regulate shoot apical meristem and floral development [54].

In *C. ensifolium*, the largest miRNA family size was miR166 which consisted of 8 members, followed by miR171 and miR396 both with 5 members. The miRNA families with a large number of members may be indicative of their function in floral development. Different family members also displayed drastically different expression levels (Additional files 2 and 3). For example, in PS1 members of the miR159 family had TPM ranging from 271.6 (cen-miR159d) to 105939.6 (cen-miR159a.1). This was also the case for some other miRNA families, such as miR166 (0 ~ 30090.4 tpm) and miR396 (23.9 ~ 6146.7 tpm). It is possible that the most abundant member of a miRNA family correlates with its importance for performing key regulatory functions at a particular flowering phase.

### Identification of novel miRNAs and ta-siRNA in *C. ensifolium* flower buds

We identified 24 novel miRNA and 21 novel miRNA* which corresponded to 28 precursor sRNA. Some members were tissue-specific miRNA, and most of these members were miRNA*, such as novel-miR02*, novel-miR10*, novel-miR20*, and novel-miR24*. The expression of these miRNA* was much lower than others which was consistent with previous reports [10, 45, 52]. As miRNA* strands are degraded rapidly during the biogenesis of mature miRNAs, it is possible that the miRNA* strand accumulates at a lower level [10].

The novel *C. ensifolium* miRNAs displayed much higher expression levels compared to conserved families. Due to the limited genomic information available for *C. ensifolium,* many of the highly expressed miRNA’s identified in this study are being reported for the first time. These miRNA may represent only a small portion of novel miRNA families involved with *C. ensifolium* floral development and the sequence information provided will be critical for future research.

Trans-acting siRNAs (ta-siRNAs) are a class of small RNAs that regulate plant development [55]. Ta-siRNAs are miRNA-triggered secondary siRNAs from miRNA-targeted noncoding transcripts. We identified two ta-siRNAs, one ta-siRNA gene was homologous to TAS3a1 and the other is a novel TAS. TAS3 is the center of an autoregulatory network involving miR390, which targets auxin response factors 2 (ARF2), ARF3, and ARF4 [56, 57]. Mi390 assists in cleaving the TAS3 transcript. TAS3 inhibits ARF2/3/4 expression, and ARF4 down regulates miR390 accumulation. In contrast, ARF3 up-regulates miR390 in response to auxin [58]. These target ARF genes, are conserved in angiosperms and play critical roles in plant development [59, 60].

### Genes targeted by miRNA and their function during *C. ensifolium* flowering

Analysis of miRNA targets revealed 47 conserved miRNAs, one new miRNA, and 790 potential targets. Most of these miRNA targeted more than one gene, suggesting these miRNA are involved in several biological processes. Similarly, a single gene was also targeted by several miRNAs. This result could be due to miRNAs mapping to the same cDNA at different sites, and cleaving the mRNA into different-sized fragments [16].

Of these target genes, 62 belonged to MADS-box genes in the expanded ABCDE model of floral development, which determines identity of flower organs [28]. As reported in other plant species, miR156 can act on squamosa promoter-binding (SPB) gene [61, 62]. The SPB promotes flowering by activating miR172 and other MADS box genes [62]. Overexpression of miR156 decreases the SPB’s expression, which results in delayed flowering [17], whereas overexpression of miR172 in Arabidopsis accelerates flowering [24, 63]. Also, miR172 was involved in determining the identity of floral organs by regulating the expression of its target AP2 genes [18, 24]. However, miR156 and miR172 appear to have opposite effects on flowering and regulating floral development in coordination with one another [45, 64]. The conserved mechanism of miR156 and miR172 which mediates transition from the vegetative to the reproductive phase is also reported in orchids *Phalaenopsis aphrodite* [20], *Erycina pusilla* [22] and *Orchis italica* [21]. In our study, cen-miR156 and cen-miR172 were identified during both flowering phases, suggesting that they may have roles in flowering and determining identity of floral organs *in C. ensifolium*. Another MADS-box gene, DEF-like transcription factor, plays a role in the diversification of tepals and lip. Here, the DEF-like gene 3 was targeted by cen-miR5179, and the cleavage was confirmed by RLM-Race. The similar result is also reported in orchid *O. italica* [21]. The regulation of miR5179 on DEF-like transcript factor suggests that miR5179 exerts an important function in the diversification of the perianth [21].

MiR159 targeted several MYB genes that act as floral developmental regulators [16, 65]. For example, miR159 degrades its target MYB33 in the process of gibberellin (GA)-induced flowering in Arabidopsis, resulting in non-expression of LEAFY, as well as delayed flowering in short days and male sterility [14, 66, 67]. MYB33 may mediate GA signaling by binding to the promoter of the floral meristem-identity gene, LEAFY [68]. In addition, MYB21, MYB24, and MYB57 are all DELLA repressible GA-response genes that mediate stamen filament growth, and regulate stamen maturation through jasmonate [69].

Xyloglucan endotransglucosylase (XTH) expression was targeted by cen-miR159f. The xyloglucan plays an important structural role in the primary and secondary walls [70]. In plant cell walls, the availability of xyloglucan and its oligosaccharides determine tissue tension and is involved in the control of plant growth and development [71, 72]. In *C. ensifolium*, XTH may play an important role in petal morphogenesis [73]. In *Arabidopsis*, XTH9 expression is very high in meristematic tissues [74]; AtXTH28 is specifically involved in the growth of stamen filaments, and is required for successful self-pollination in certain flowers [75].

In addition, 21 target genes were involved in various hormone signaling pathways. As shown in our results, three members of cen-miR167 family were all expressed at low levels during both flowering phases and targeted auxin response factor. In Arabidopsis, miR167 targets two members (ARF6 and ARF8) of auxin response factor (ARF) family, which regulate gynoecium and stamen development in immature flowers [76, 77]. The antagonistic effects of the two hormones, auxin and ethylene, control abscission of flowers [78]. In low concentrations, auxin can delay the senescence of flowers, and the biogenesis of ethylene [79]. Notably, in previous studies on cymbidium, flower abscission is not sensitive to ethylene, although ethylene-sensitive flower abscission is very common in other plant species [80].

Additionally, 134 target genes did not match any homologs in the database, and many of these genes were annotated with only vague information i.e. unnamed protein product, predicted protein etc. This may be due to the limited genomic information available for Cymbidium.

### Differential expression of miRNAs during *C. ensifolium* floral development

MiRNAs that regulate flowering phenology during floral development have been previously reported [18, 81]. In this study, seven miRNA showed significantly different expression between two flowering phases. This was confirmed by RNA-seq and RT-qPCR. The results indicate these miRNA play different roles during floral development. Among the seven miRNA, three of them, cen-miR394, cen-miR160a-5p, and cen-miR535-5p, were significantly up-regulated at PS4, while the other four miRNA, novel-miR37, novel-miR11, novel-miR31 and cen-miR396a-5p, were down-regulated at PS4. In GO analysis, the genes targeted by differently expressed miRNA were significantly enriched within “multicellular organismal development” and “RNA binding” in term of “biological process” and “molecular function”, respectively. The results suggested these target genes could be floral development genes and transcription factors. In previous studies, miR394 and its target, an F-box gene referred to as Leaf Curling Responsiveness (LCR), were reported to be involved in the regulation of leaf curling-related morphological development [82]. In Arabidopsis, miR396 can regulate the expression of Growth Regulating Factor (GRF) genes, which are known to be involved in the control of cell proliferation during leaf and root development [83]. MiR160 negatively regulates ARF10, ARF16, and ARF17 [84, 85]. A loss-of-function mutant floral organs in carpel (foc) exhibits some defects of flowering, such as the formation of irregularly shaped flowers and floral organs inside siliques, as well as reduced fertility [84, 85]. In *C. ensifolium*, miR396a, miR394, and miR160 may also be involved in floral cell proliferation and morphological development.

## Conclusion

This study established a miRNA database for *C. ensifolium*, which includes the identification of conserved and novel miRNAs. All of these miRNAs were reported for the first time in cymbidium. When the miRNA was compared to the *C. ensifolium* transcriptome, we investigated a number of transcriptomic unigenes targeted by miRNAs. Some unigenes included MADS-box genes, which are known to determine the identity of flower organs, and were targeted by miR156, miR172 and miR5179. Other unigenes affect various hormones signaling pathways involved in floral development, and were targeted by miR159. Expression analysis of RNA-seq and qRT-PCR identified novel and conserved miRNA that were differentially expressed during flowering phases. They also targeted flowering related genes, such as ARF and growth regulating factor (GRF). These results suggest that these miRNAs may play a crucial role in cymbidium flowering, through regulating the expression level of flowering related genes. Identification of these miRNAs and their targets provides new insight into gene regulation of cymbidium floral development. More novel miRNAs will be identified and their biological function will be revealed as the genomic sequences become available and transformation is established in cymbidium.

## Additional files

Additional file 1:
**Real-time quantitative PCR primer sequences for candidate miRNAs.**
Additional file 2:
**The conserved miRNA family among all of the species.**
Additional file 3:
**The graphic display of conserved miRNA family among plant species.**
Additional file 4:
**The expression of the novel miRNA in four libraries from two flowering phases.**
Additional file 5:
**Annotation of unigenes targeted by miRNA.**
Additional file 6:
**The comparison of seven differentially expressed miRNA between RT-qPCR and RNA-seq.**
Additional file 7:
**Coefficient analysis of fold change data between RT-qPCR and RNA-seq.** Based on RNA-seq results, seven significant differentially expressed miRNA were subject to RT-qPCR assay. Data indicating relative transcript level from RT-qPCR are means of three replicates, and data from RNA-seq are means of two replicates. Scatterplots were generated by the log2 expression ratios from qPCR Log2(PS4/PS1) (x-axis) and RNA-seq Log2(PS4/PS1) (y-axis). Pearson correlation coefficient (r) was 0.948 (P < 0.01). Additional file 8:
**Gene ontology (GO) enrichment of the target genes.** The genes were targeted by differently expressed miRNA between two floral development phases in *C. ensifolium*. Additional file 9:
**The KEGG pathway enrichment of the target genes.** The genes were targeted by differently expressed miRNA between two floral development phases in *C. ensifolium.*
