# Supplementary figures and images for: Characterization and comparative profiling of the small RNA transcriptomes in two phases of flowering in Cymbidium ensifolium
Source: BMC Genomics. 2015 Aug 20;16(1):622. doi: 10.1186/s12864-015-1764-1 (PMC4546042; doi:10.1186/s12864-015-1764-1)

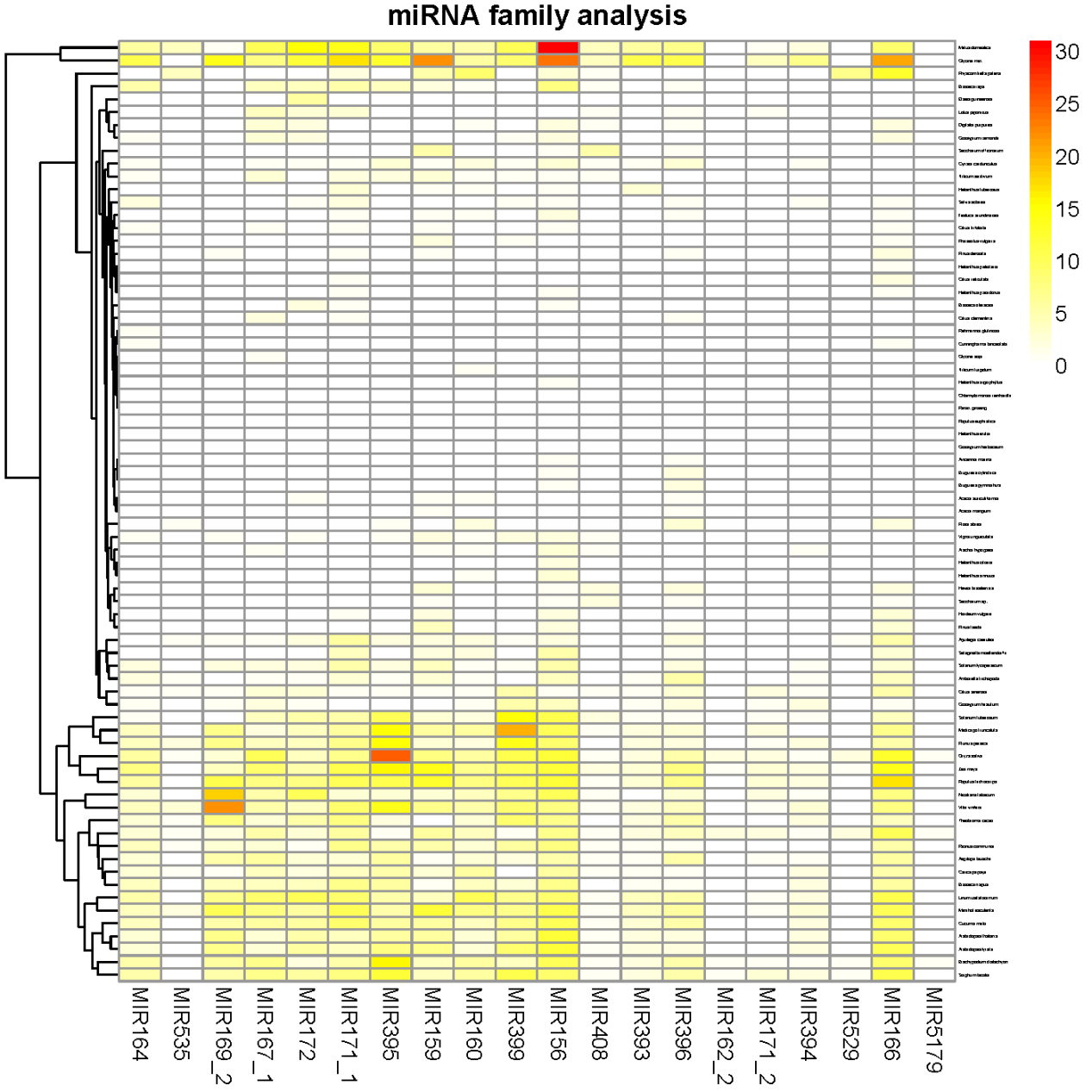


The number of miRNA family members present

Supplement: Additional file 3: — The graphic display of conserved miRNA family among plant species. [file 12864_2015_1764_MOESM3_ESM.docx]
