# Supplementary material for: Characterization and comparative profiling of the small RNA transcriptomes in two phases of flowering in Cymbidium ensifolium
Source: BMC Genomics. 2015 Aug 20;16(1):622. doi: 10.1186/s12864-015-1764-1 (PMC4546042; doi:10.1186/s12864-015-1764-1)
Supplement: Additional file 7: — Coefficient analysis of fold change data between RT-qPCR and RNA-seq. Based on RNA-seq results, seven significant differentially expressed miRNA were subject to RT-qPCR assay. Data indicating relative transcript level from RT-qPCR are means of three replicates, and data from RNA-seq are means of two replicates. Scatterplots were generated by the log2 expression ratios from qPCR Log2(PS4/PS1) (x-axis) and RNA-seq Log2(PS4/PS1) (y-axis). Pearson correlation coefficient (r) was 0.948 (P < 0.01). [file 12864_2015_1764_MOESM7_ESM.docx]

RNA-seq Log2(PS4/PS1)

RT-qPCR Log2(PS4/PS1)
